# Expression of constitutively active erythropoietin receptor in pyramidal neurons of cortex and hippocampus boosts higher cognitive functions in mice

**DOI:** 10.1186/1741-7007-9-27

**Published:** 2011-04-28

**Authors:** Derya Sargin, Ahmed El-Kordi, Amit Agarwal, Michael Müller, Sonja M Wojcik, Imam Hassouna, Swetlana Sperling, Klaus-Armin Nave, Hannelore Ehrenreich

**Affiliations:** 1 Division of Clinical Neuroscience, Max Planck Institute of Experimental Medicine, Hermann-Rein-Str.3, 37075 Göttingen, Germany; 2 DFG Research Center for Molecular Physiology of the Brain (CMPB), Humboldtallee 23, 37073 Göttingen, Germany; 3 Department of Neurogenetics, Max Planck Institute of Experimental Medicine, Hermann-Rein-Str.3, 37075 Göttingen, Germany; 4 Department of Neuro- and Sensory Physiology, Georg-August-University, Humboldtallee 23, 37073 Göttingen, Germany; 5 Department of Molecular Neurobiology, Max Planck Institute of Experimental Medicine, Hermann-Rein-Str.3, 37075 Göttingen, Germany

## Abstract

**Background:**

Erythropoietin (EPO) and its receptor (EPOR) are expressed in the developing brain and their transcription is upregulated in adult neurons and glia upon injury or neurodegeneration. We have shown neuroprotective effects and improved cognition in patients with neuropsychiatric diseases treated with EPO. However, the critical EPO targets in brain are unknown, and separation of direct and indirect effects has remained difficult, given the role of EPO in hematopoiesis and brain oxygen supply.

**Results:**

Here we demonstrate that mice with transgenic expression of a constitutively active EPOR isoform (cEPOR) in pyramidal neurons of cortex and hippocampus exhibit enhancement of spatial learning, cognitive flexibility, social memory, and attentional capacities, accompanied by increased impulsivity. Superior cognitive performance is associated with augmented long-term potentiation of cEPOR expressing neurons in hippocampal slices.

**Conclusions:**

Active EPOR stimulates neuronal plasticity independent of any hematopoietic effects and in addition to its neuroprotective actions. This property of EPOR signaling should be exploited for defining novel strategies to therapeutically enhance cognitive performance in disease conditions.

## Background

The hematopoietic growth factor erythropoietin (EPO) increases erythrocyte numbers by preventing apoptosis of erythroblasts in the bone marrow. Recombinant human EPO (rhEPO) is a clinically widely used drug, approved for the treatment of anemia worldwide. The surprising clinical observation that rhEPO improves cognitive function has always been attributed to the increase in hemoglobin levels and thus enhanced tissue oxygenation (for review see [1-3]). Even after the discovery of EPO and EPO receptor (EPOR) in the brain [4,5], it took years until direct EPO effects on the central nervous system were first explored by *in vivo *experiments [6]. In the following, EPO turned out to have potent antiapoptotic, antioxidative and anti-inflammatory properties in the brain (for review see for example [7,8]). Downstream signaling pathways of EPO in cells of the nervous system were extensively explored, showing an involvement of signal transducers and activators of transcription (STATs), phosphatidylinositol-3 kinase (PI3K)/AKT, RAS/extra-cellular signal-regulated kinase (ERK1/2), nuclear factor kappa B (NF-kappa B) and calcium [9-11]. A large number of preclinical studies followed, devoted to the employment of EPO as a neuroprotective agent (for review see [12]). Recent clinical trials on patients with schizophrenia [13,14] or chronic progressive multiple sclerosis [15] as well as a trial involving extremely preterm infants [16], which all demonstrated improved cognitive outcome upon EPO treatment, strongly suggested that this growth factor should be considered as a candidate neuroprotective drug counteracting cognitive decline. Nevertheless, the relevant mechanisms of action remained unclear and difficult to formally separate from blood oxygenation effects.

As compared to neuroprotection studies in disease models (for review see [12]), work on the function of the EPO system in normal brain is scarce. Based on the prominent effects of EPO on cognition, we hypothesized that an important physiological role of EPO in postnatal life or adulthood might be the modulation of neuroplasticity and of higher cognitive functions. We showed previously that in healthy young mice high-dose EPO treatment over three weeks enhanced hippocampal long-term potentiation (LTP) and memory [17], as well as executive and attentional functions [18]. A recent functional magnetic resonance imaging (fMRI) study on healthy volunteers reported enhancement of the hippocampal response during memory retrieval after only one high dose of EPO [19]. In all of these cases, EPO was applied peripherally, penetrated an intact blood-brain-barrier [20-22], and likely bound to all major classes of brain cells expressing EPOR, making it impossible to delineate the cell type(s) responsible for enhanced cognition.

To explore whether active EPOR in cortical neurons has a direct impact on cognitive functions in the non-injured brain, we chose a transgenic strategy. To be independent of rhEPO and to genetically define the neuronal target cells, we expressed a constitutively active form, EPOR^R129C ^in the postnatal mouse forebrain, using a transgene driven by the α-calcium/calmodulin-dependent protein kinase II (α-CaMKII) promoter. The substitution in EPOR^R129C ^(cEPOR) confers growth factor-independent survival and tumorigenicity to hematopoietic Ba/F3 cells that are normally dependent on interleukin-3 for growth and survival [23,24]. The constitutive activity results from the formation of a disulfide-linked receptor homodimer, mimicking the EPO-bound form [25]. Expression of cEPOR thus activates EPOR signaling without requirement of a ligand [26].

We show here that cEPOR expression in pyramidal neurons of cortex and hippocampus of transgenic (TG) mice prominently enhances higher cognitive performance. Superior cognition is correlated with enhanced paired-pulse facilitation and long-term potentiation at the Schaffer collateral CA1 synapse, indicative of increased short- and long-term plasticity. Cognitive augmentation in this genetic model is different from that observed in mice receiving rhEPO injections and comes at the price of higher impulsivity and reduced behavioral control under strong cognitive challenge. We conclude that active EPOR stimulates neuronal plasticity independent of any hematopoietic effects and in addition to its neuroprotective properties.

## Results

### Transgenic cEPOR expression in cortical and hippocampal neurons

**Figure 1:**
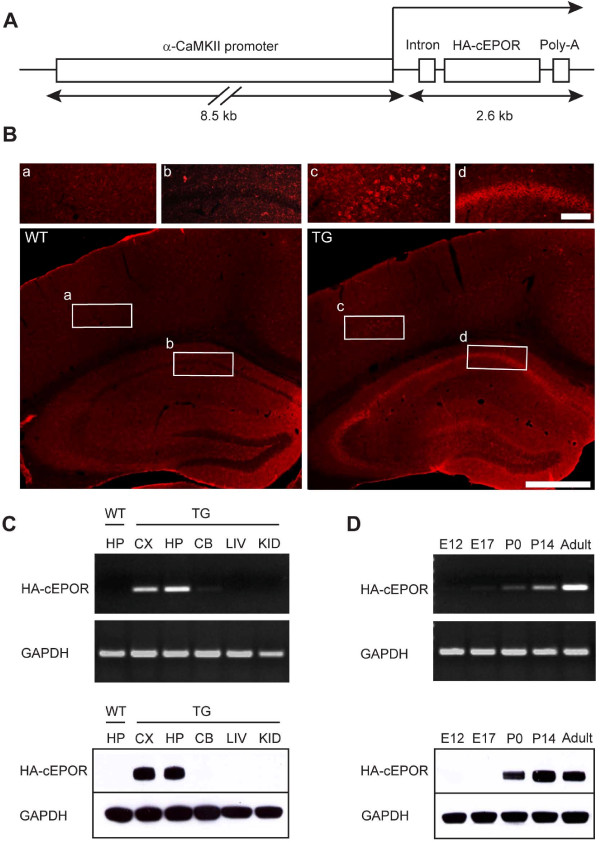
**Construction and characterization of HA-cEPOR TG mice *('TG1')***. **(A) **Construct HA-cEPOR used for production of HA-cEPOR TG mice. HA-cEPOR, flanked by a hybrid intron at the 5' end and a polyadenylation signal at the 3' end was placed under the control of the α-CaMKII promoter. **(B) **Forebrain-specific expression of HA-cEPOR transgene revealed by immunohistochemistry. A monoclonal antibody against the HA-tag was used to stain coronal sections of the hippocampus. Expectedly, HA-cEPOR expression is absent in WT mice [**(a) **and **(b) **are magnifications of the respective regions of interest]. In TG mice, HA-cEPOR expression is restricted to pyramidal neurons of the cortex **(c)**, CA1 **(d) **as well as CA3 subregions of the hippocampus and granular layer of the dentate gyrus. Scale bars; 100 μm and 500 μm. **(C) **Tissue-specific expression of HA-cEPOR mRNA (top) and protein (bottom) in TG mice. TG mRNA expression was detected by PCR using TG specific primers, yielding a 362 bp product. Western blot analysis of HA-cEPOR using a monoclonal antibody against HA-tag revealed a 64 kDa band. HA-cEPOR mRNA and protein were expressed in cortex (CX) and hippocampus (HP) of TG mice but not in cerebellum (CB) or in peripheral tissues (LIV: liver; KID: kidney). GAPDH was used as the internal control for both mRNA (431 bp) and protein (36 kDa) expression analysis. **(D) **Developmental regulation of the HA-cEPOR transgene. HA-cEPOR transgenic mRNA and protein expression was not seen in fetal tissue ('embryonic' day 12 and 17), but detected at early postnatal days (P0, P14) and remained constant until adulthood.

To systematically investigate the role of the EPO system in learning, memory and attention, we generated a TG mouse line expressing a constitutively active form of EPOR (cEPOR) under control of the α-CaMKII promoter (Figure 1A), which restricts expression of cEPOR to forebrain pyramidal neurons of postnatal mice. A hemagglutinin (HA) tag at the amino terminus of cEPOR allowed monitoring of transgene expression. Of three independent lines produced, we analyzed two (TG1 and TG2) in detail, which showed a similar spatio-temporal expression pattern of the mutant receptor (Figure 1B and Additional file 1). In fact, cEPOR expression was enriched in cortex and hippocampus (Figure 1B, C) but not in cerebellum and peripheral tissues (Figure 1C). Western blot analysis demonstrated the presence of a 64 kDa mutant receptor in the brains of TG animals that was regulated developmentally over time and augmented in early postnatal days (Figure 1D). Mice lacked any signs of neuropathology, were long-lived, and reproduced well. Animals in both lines showed normal growth and development, body weight, mating behavior and gross brain morphology, virtually identical to that of WT mice (see Additional file 2). Moreover, hematocrit levels were comparable between WT and TG mice (WT mice: 45.00 ± 0.3162%, N = 5; TG mice: 46.00 ± 1.080%, *P *= 0.36; N = 4). Thus, behavioral differences would not be secondary to developmental changes of the brain or hyperoxygenation.

### Slight hyperactivity but normal basic behavior of cEPOR TG mice

**Figure 2:**
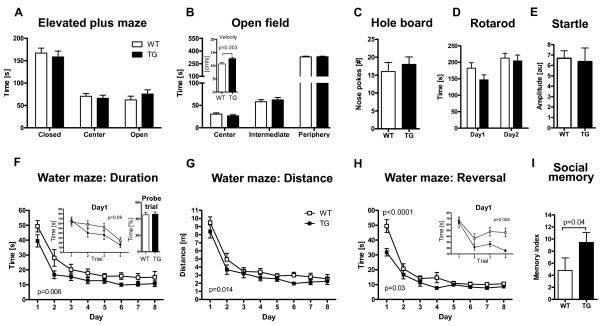
**Essentially normal basic behavior of cEPOR TG mice contrasts the distinctly superior cognitive performance**. TG mice were normal in basic behavioral functions **(A - E) **except for slight hyperactivity, as demonstrated by increased velocity in the open field **(inset B)**. In basic cognitive tasks, that is spatial learning/memory and cognitive flexibility as evaluated by Morris water maze **(F - H) **and social memory **(I)**, cEPOR TG mice were significantly superior to WT. WT n = 18; TG n = 13; mean ± SEM presented. **Insets in F**: within - day learning on day one of the hidden platform task: all four trials presented; probe trial expressed as % of time spent in target quadrant; Inset in **H**: within-day learning on day one of the reversal task: all four trials presented.

We first assessed basic behavioral functions, such as locomotor and exploratory activity, anxiety, and motor performance, in cEPOR TG mice in comparison to WT littermates. In the elevated plus maze, there was the expected significant effect of arms (2-way ANOVA, F(2,90) = 66.70; *P *< 0.0001), but neither a genotype (*P *= 0.9997) nor an interaction effect (*P *= 0.4748), indicating comparable anxiety levels between TG and WT mice (Figure 2A). In the open field test, mice did not differ with respect to the time spent in zones (2-way ANOVA, effect of genotype *P *= 0.9996) (Figure 2B). However, TG mice showed increased velocity (Mann-Whitney U-test, *P *= 0.003) (Figure 2B inset) as well as distance travelled (data not shown). There were no differences between groups in exploratory activity, locomotor coordination, motor learning and acoustic startle response (Figure 2C,D,E).

### Enhanced spatial learning and cognitive flexibility

Morris water maze, a sensitive test for hippocampus-dependent learning/memory processes [27], yielded comparable performance of TG and WT in the visible platform task (data not shown). In contrast, in the hidden platform task, a significant effect of days (2-way ANOVA for repeated measures F(7,203) = 24.66; *P *< 0.0001) as well as a significant effect of genotype (2-way ANOVA for repeated measures, F(1,29) = 8.863; *P *= 0.006) and no interaction effect (*P *= 0.87) were observed (Figure 2F). The tendency of a different starting point on day one of the hidden platform task made us analyze the four trials of this day separately. The pattern obtained explains the tendency, even though 2-way ANOVA for repeated measures failed to yield a significant genotype effect of the within-day curve (*P *= 0.09; inset Figure 2F). This pattern of faster within-day learning is lost with increased overall level of performance (see Additional file 3). To exclude hyperactivity as a potential confounding variable, we evaluated the distance travelled for locating the hidden platform, a parameter independent of swimming speed. Again, there was a significant effect of genotype (2-way ANOVA for repeated measures, F(1,29) = 6.791; *P *= 0.014; Figure 2G), indicating enhanced hippocampus-dependent learning and memory function in cEPOR TG mice. The similar performance level of both groups on day eight of the hidden platform paradigm (*P *= 0.8) may explain the negative result in the probe trial (inset Figure 2F) and allowed us to further assess cognitive flexibility of cEPOR TG mice using the reversal paradigm. Again, 2-way ANOVA for repeated measures revealed a significant effect of days (F(7,196) = 55.71, P < 0.0001), significant effect of genotype (F(1,28) = 5.090, *P *= 0.03) and a significant interaction effect (F(7,196) = 3.649, *P *= 0.001). Post-hoc analysis indicated a significant difference on day one (Bonferroni post-hoc test, *P *< 0.0001). To further delineate performance of both groups on this critical day one, we applied a 2-way ANOVA for repeated measures over the four trials of this day. Both groups significantly improved over time (F(3,84) = 12.71, *P *< 0.0001). However, there was a significant genotype effect (F(1,28) = 9.632, *P *= 0.004), pointing to faster adaptation of cEPOR TG mice to the new platform position (Figure 2H and inset).

### Enhanced synaptic plasticity of the hippocampus

**Figure 3:**
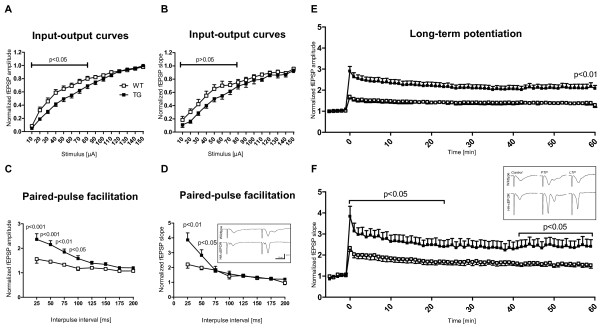
**cEPOR mice reveal improved synaptic short- and long-term plasticity**. **(A,B) **Input-output curves of evoked EPSPs: The moderate right-shift of response amplitudes **(A) **and slopes **(B) **in cEPOR mice suggests slightly reduced basal excitability (WT n = 10, TG n = 10). **(C,D) **Short-term plasticity as tested by paired-pulse facilitation was markedly enhanced in cEPOR mice. The amplitude **(C) **and slope **(D) **of the second EPSP are normalized to the first EPSP of a given twin pulse. Plotted are the averages of 12 (wildtype) and 11 (cEPOR TG) slices. Error bars represent SEM and the level of significance of the changes observed is indicated. The sample responses shown in the inset **(D) **were elicited by 30 μA stimuli separated by 25 ms; stimulation artefacts are truncated. Baseline stimulation intensities (adjusted to obtain about half maximum fEPSP amplitudes) for stimulation in PPF and long-term potentiation (LTP) experiments differed only slightly among genotypes. On average, the chosen stimulation intensities were 50 μA for WT and 60 μA for transgenic slices. **(E,F) **Also, post tetanic potentiation (PTP) as well as LTP was more pronounced in cEPOR mice. LTP was induced at time 0; plotted are the normalized averages of 11 slices for each genotype. The sample responses in the inset **(F) **show EPSPs under baseline conditions, immediately after the LTP-inducing stimulus, that is the phase of PTP, and 60 minutes after stimulation. Responses were elicited by 60 μA stimuli, scaling is identical to panel D.

We hypothesized that the improvements of hippocampal learning are associated with a higher degree of synaptic plasticity. To assess changes in synaptic function and plasticity at the Schaffer collateral-CA1 synapse, extracellular field potential recordings were performed in *stratum radiatum *of the CA1 subfield of acute hippocampal tissue slices. Basal synaptic function and neuronal excitability were judged on the basis of input-output curves and synaptic plasticity was tested by paired-pulse stimulation and long-term potentiation (LTP)-inducing trains of tetanic stimuli. The absolute amplitudes and slopes of orthodromically evoked field excitatory postsynaptic potentials (fEPSPs) did not differ significantly among WT and TG mice. However, the normalized input-output curves (10-150 μA stimuli) were slightly right-shifted for cEPOR mice at low stimulation intensities (≤70 μA, Figure 3A,B). On average, half maximum response amplitudes were obtained with 30 μA and 50 μA stimuli in WT (n = 14 slices) and TG mice (n = 12 slices), respectively.

Clear differences were observed in response to twin-pulse stimulation. In cEPOR TG mice, paired-pulse facilitation with interpulse durations of up to 150 ms was significantly increased as compared to WT mice (Figure 3C,D). At the shortest interpulse interval tested (25 ms), the fEPSP amplitudes increased by 59.3 ± 15.5% and 127.9 ± 22.7% for WT (n = 12 slices) and TG mice (n = 11), respectively, and fEPSP slopes increased by 113.6 ± 20.2% and 268.2 ± 45.4%.

LTP-inducing stimuli (three trains of 100 Hz, each lasting for one second) also resulted in a more pronounced potentiation of fEPSPs in cEPOR mice. This augmentation was already evident during the initial phase of LTP right after high frequency stimulation and remained stable for the following 60 minutes analyzed, that is the phase considered as early LTP (Figure 3E,F). Immediately after tetanic stimulation, that is the phase being referred to as post-tetanic potentiation (PTP), fEPSP amplitudes were increased by 68.6 ± 9.4% and 190.5 ± 22.4% for WT and TG mice, respectively, and fEPSP slopes increased by 133.3 ± 16.0% and 283.3 ± 47.9% (Figure 3F). This initial post-tetanic potentiation decayed over the course of three to five minutes and the evoked responses stabilized. One hour after LTP induction, fEPSP amplitudes were still increased by 38.4 ± 7.4% and 116.7 ± 18.7% and fEPSP slopes were elevated by 51.0 ± 13.2% and 140.7 ± 32.5% (Figure 3F).

### Social memory, attention and higher cognitive capacities

Would EPO-EPOR signaling also affect higher cortical functions that cannot be easily correlated with electrophysiological readouts? We first addressed social functions in cEPOR TG mice. While there were no differences in social interaction and social approach (data not shown), cEPOR TG mice showed enhanced social memory (*P *= 0.04, Figure 2I). This behavior was not due to increased exploratory activity, since performance in the hole board test did not differ between groups (Figure 2C). Additionally, no differences in olfaction were detected between groups (data not shown) indicating that this finding reflects improved memory function in the area of social interaction.

**Figure 4:**
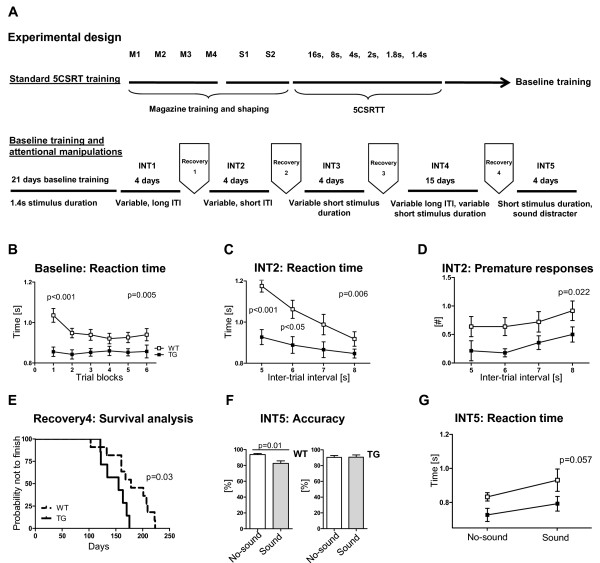
**cEPOR TG mice perform better in the attentional interventions of the 5-choice serial reaction time task (5-CSRTT)**. **(A) **Experimental design depicting the standard baseline training steps in 5-CSRTT as well as attentional manipulations. **(B,C) **cEPOR TG mice showed better attentional performance reflected by shorter reaction times. **(D) **They displayed less premature responses under lower cognitive demands and **(E) **were overall faster in progressing through the consecutive training steps. **(F,G) **Additionally, they were less distractible and more resistant towards irrelevant competing auditory stimuli. WT n = 9 to 11; TG n = 7; mean ± SEM presented; INT = intervention; ITI = inter-trial interval.

We next assessed attention and behavioral control in these mice using the 5-choice serial reaction time task (5-CSRTT) (Figure 4A and Additional file 4) In this test, mice are trained on an everyday basis over many months to adequately respond to a short (≤1.4 second) light stimulus. Light stimuli appear pseudorandomly in one of five equally distant stimulus holes. Mice are only rewarded if they make a nose poke in the location where the light shows up. To be considered correct, responses have to occur in a short limited-time window after stimulus presentation. After reaching stable performance, this trained behavior is challenged via different interfering manipulations (for example sound distracters, changing stimulus durations and so on).

Training under baseline conditions for 21 days revealed enhanced attentional abilities in cEPOR mice demonstrated by significantly lower reaction times (2-way ANOVA for repeated measures, effect of genotype F(1,16) = 10.73, *P *= 0.005, Figure 4B). Post-hoc analysis indicated a significant difference in the first trial block (that is the first ten trials collapsed over 21 days) (*P *< 0.001). This pattern remained robust even in face of higher cognitive challenges in intervention phase 2 (INT2) (effect of genotype F(1,14) = 10.42, *P *= 0.006; effect of inter-trial interval (ITI) F(3,42) = 19.83, *P *< 0.0001; effect of interaction F(3,42) = 5.455), *P *= 0.003, Figure 4C). Post-hoc evaluation revealed significantly lower reaction times in cEPOR TG mice in ITI5 and ITI6 (*P *< 0.001 and *P *< 0.05, respectively). Furthermore, cEPOR TG mice tended to show less premature responses in INT2 compared to WT littermates (effect of genotype F(1,14) = 6.658, *P *= 0.022, Figure 4D). Since progression until 'Recovery4' is performance-dependent, unifying various aspects of learning and cognitive control, we analyzed the proportion of mice finishing this phase until experimental day 250. Survival analysis showed a significant difference between groups (*P *= 0.03), indicating overall faster task progression in cEPOR TG mice (Figure 4E).

### Resistance to behavioral distracters

Having obtained a consistently superior performance of TG mice in the whole series of consecutive 5-CSRTT attentional challenges, we exposed the mice to interfering acoustic stimuli. In the intervention phase 5 (INT5), mice were randomly confronted either with trials, where a sound distracter was simultaneously applied with a short light stimulus, or with trials lacking such distracting auditory stimulus. The effect of the sound distracter on attentional accuracy was investigated. In cEPOR TG mice, the sound distracter did not affect attentional accuracy (Wilcoxon Test, *P *= 0.94) in contrast to WT littermates where sound distraction led to a decrease in accuracy (*P *= 0.01, Figure 4F). Reaction times showed here only borderline significance (effect of genotype *P *= 0.057). Sound distraction led to a similar increase in reaction times in both groups (effect of sound F(1,14) = 6.572, *P *= 0.023, Figure 4G).

### Increased impulsivity and reduced behavioral control under challenge

**Figure 5:**
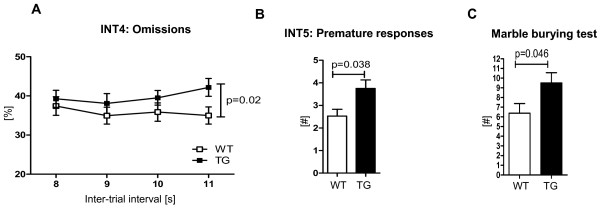
**cEPOR TG mice show increased impulsivity and reduced behavioral control under conditions of high cognitive challenge**. **(A) **cEPOR TG mice were more distractible in trials comprising low frequency stimulus presentations (longer inter-trial interval) and **(B) **more impulsive in sessions including an irrelevant auditory distracter. **(C) **Additional evaluation of these phenomena using the marble burying test confirmed the impulsive phenotype. For all figures WT n = 9; TG n = 7, except **(C)**: WT n = 18; TG n = 12; mean ± SEM presented.

Experimental cEPOR transgene expression was not ubiquitous in all EPO responsive cells in the brain, but restricted to forebrain pyramidal neurons, suggesting that a natural balance of stimulatory and inhibitory circuits may have been perturbed. We therefore wondered whether overall superior cognitive performance would come at a 'price' and searched for subtle behavioral defects. Indeed, an interesting behavioral abnormality was impulsivity. Under conditions of long inter-trial intervals (11 seconds) in combination with variable, short stimulus duration, cEPOR TG mice tended to have more omissions compared to WT littermates (Mann-Whitney U-test, *P *= 0.02, Figure 5A). In contrast to their resistance towards attentional distractions, cEPOR TG mice had more premature responses in the task where they were confronted with sound distracters, that is responses that occurred before the light-coupled sound appeared (Mann-Whitney U-test, *P *= 0.038, Figure 5B). To address whether signs of increased impulsivity are also detectable in simpler readouts of this behavioral feature, we performed the marble burying test. Indeed, cEPOR TG mice buried more marbles compared to WT littermates (Mann-Whitney U-test, *P *= 0.046, Figure 5C). Thus, confining enhanced EPOR activity to glutamatergic cortical projection neurons in the reported experiment may have specific disadvantages, and it will be interesting to compare the here obtained behavioral pattern with the *in vivo *effects of enhanced EPO-EPOR signaling in other neuronal subpopulations.

## Discussion

In order to investigate how the EPO system influences cognitive performance and synaptic plasticity, and to experimentally prove that previously reported effects of rhEPO on cognition in patients are independent of EPO effects on hematopoiesis/brain oxygen supply, we created a novel mouse model with constitutive EPOR expression in cortical and hippocampal neurons that are defined by α-CaMKII promoter activity. This way, we were able to specifically mimic EPO system function independent of any ligand in pyramidal neurons of cortex and hippocampus, that is regions pivotal for learning and memory processes. In other words, we 'over-accentuated' the endogenous action of the EPO system in specific cortical and hippocampal layers, particularly the CA1 subregion [28], to delineate the contribution of these neuronal subpopulations to the EPO effects on cognition. We found that selective constitutive expression of EPOR in forebrain neurons leads to a phenotype with superior performance in higher cognitive tasks. Behaviorally, this phenotype is accompanied by slightly increased activity and impulsivity. Electrophysiologically, both short- and long-term plasticity at the Schaffer collateral CA1 synapses are significantly increased in cEPOR expressing TG mice.

Field potential recordings revealed augmented paired-pulse facilitation, short-term potentiation and LTP in cEPOR TG mice. One possible cause for the differences observed in the twin-pulse stimulation results might be the slight right shift of the input-output curves which suggests somewhat reduced baseline excitability and thus leaves more room for potentiated response. Even though the detailed underlying molecular mechanisms cannot be determined on the basis of our extracellular recordings, the enhanced paired-pulse facilitation confirms that an increased number of transmitter quanta can be released in cEPOR TG mice (for review see [29]). Accordingly, the dynamic range of synaptic plasticity (and hence efficacy) is clearly extended as compared to WT mice.

The gain in paired-pulse facilitation is comparable to changes observed earlier in mice receiving EPO injections [17]. However, in cEPOR TG mice, LTP stabilized at higher levels. Since both paired-pulse facilitation and LTP are augmented, both pre- and postsynaptic mechanisms seem to contribute to the improved synaptic plasticity. Upon high frequency stimulation (LTP induction), the immediate phase of post tetanic potentiation is known to be independent of kinase activity [30]. The following 60 minutes of LTP reflect 'early LTP', which at the Schaffer collateral CA1 synapse is NMDA receptor dependent, involves activation of various kinases leading to AMPA receptor phosphorylation, but is independent of protein synthesis and/or gene transcription [30,31]. Interestingly, some of the kinases involved in the phosphorylation events during early LTP (MAPK, PI3K and ERK) are indeed part of the EPO signaling cascades [10] and thus might constitute putative sites of signal convergence. Nevertheless, the enhanced spatial learning and cognitive flexibility observed in cEPOR TG mice suggest that, even though not evaluated electrophysiologically, the late phase of LTP, that is the phase requiring gene transcription and protein synthesis, is augmented as well [30,31].

A detailed and comprehensive behavioral-cognitive analysis of cEPOR TG mice was performed to demonstrate that increased EPO signaling in cortex and hippocampus enhances a whole array of learning and memory processes, as well as cognitive flexibility and attentional capacities, reflected by shorter reaction times and reduced distractibility through competing irrelevant auditory stimuli. Very similar higher cognitive tasks were found improved in human patients upon several months of weekly high-dose intravenous EPO treatment [13-15], pointing to specific targets of EPO action on cognition that are common to both mice and humans. Augmented EPOR signaling in cEPOR TG mice also improved social memory, which is partly dependent on hippocampal functions [32]. We note that a recent study reported on better facial recognition performance in patients with major depression following high-dose EPO application [33], supporting social cognition as another selective target of EPO effects across species.

It is important to point out that there are clear differences between EPO effects on higher cognition upon systemic administration to healthy mice [18] as compared to the selective and specific stimulation of the EPO system in forebrain pyramidal neurons reported here. In contrast to mice receiving intraperitoneal EPO injections [18], the cEPOR TG mice did not show improved performance in the (still relatively basic) initial 5-CSRTT training phases. Their superiority, however, was pronounced in the highest cognitive challenge tasks, demanding tremendous attentional capacities.

Surprisingly, under cognitively most challenging conditions, cEPOR TG mice demonstrated more premature responses as readout of impaired behavioral impulse control [34]. The slightly hyperactive and impulsive phenotype of cEPOR TG mice was further confirmed by a simple additional assay - the marble burying test. Behavioral consequences of this kind were not noted upon high-dose EPO treatment where the cellular target is defined by the almost ubiquitous presence of EPOR throughout the brain. A potential explanation for the cEPOR TG phenotype of impulsivity and hyperactivity might be the continuous stimulation of the EPO system exclusively in cortical projection neurons. Since the frontal cortex has reciprocal projections to subcortical and basal brain regions [for example [35-37]], responsible for locomotion, motivation and impulsivity (for review see for example [38-40]), the excitation of frontal pyramidal neurons might lead to a relative deficit in the simultaneous inhibitory regulation of these areas, consistent with a disturbance of the homeostatic balance within neuronal networks, resulting in impulsivity and hyperactivity of TG mice. This hypothesis is presently under systematic investigation in our laboratory through selected cEPOR expression in subpopulations of inhibitory interneurons.

Interestingly, there is an ongoing debate regarding the nature of brain EPOR. The fact that non-hematopoietic but neuroprotective EPO variants have been identified makes the additional existence of a different EPOR in the brain very likely (for review see [7,8]). Indeed, the work of Xiong and others [41,42] may well be interpreted along these lines. These authors demonstrated in a traumatic brain injury model worse outcome of neural EPOR-deficient mice, but, surprisingly, beneficial effects of EPO treatment even in the absence of the 'classical' neural EPOR. Moreover, they found that EPOR null mice *per se *are not impaired in spatial learning, indicating that the 'classical' brain EPOR may not be crucial for this task. This again emphasizes that the superior performance of cEPOR TG mice in spatial learning and memory, induced by over-activation of one selected population of neurons only, is partly explained by the provoked dysbalance in neuronal networks. In fact, some of the cognitive phenotypes observed here may also be derived from over-activation of downstream signaling cascades in neurons which are not endogenously triggered by EPO, thereby creating a somewhat 'artificial' scenario. Nevertheless, the cEPOR approach taken here may ultimately help to delineate the role of discrete neuronal subpopulations in cognitive processes.

EPO and EPOR are expressed at very high levels in the developing central nervous system [43-47]. In contrast, their expression is markedly reduced postnatally and remains low in the normal adult brain [48]. Both genes are upregulated under disease conditions in various different cell types in the brain, possibly to exert neuroprotective effects [49,50]. Our present data indicate that activated EPOR serves a role in neuroplasticity, independent of and in addition to its anti-apoptotic neuroprotective tasks. Since the steady-state level of both receptor and ligand is nevertheless low in the uninjured brain, we suggest that this function may also be disease-relevant. We propose a model in which EPO-EPOR induction under disease conditions not only prevents neuronal cell death, but also triggers the enhanced neuronal plasticity that is required to functionally compensate for lost neuronal functions. It is intriguing that this could indicate a strategy of the neocortex, which is known to provide striking functional compensations after injury.

## Conclusions

This study, together with our previous work, strongly supports a biological role for EPO in cognitive processes. The potential of this role should be exploited to define novel strategies to therapeutically enhance brain plasticity and cognitive performance in disease conditions.

## Methods

### Generation and characterization of transgenic (TG) mice

EPOR^R129C ^(cEPOR) bears a single point mutation at nucleotide 484, that is in the exoplasmic domain, causing a substitution of cysteine for arginine at codon 129 of the N terminus (R129C). The cDNA sequence of cEPOR, containing a hemagglutinin (HA; YPYDVPDY) tag inserted five residues downstream of the signal peptidase cleavage site [51], was excised with PacI and SalI from the pMX-HA-cEPOR plasmid. The HA-cEPOR cDNA was inserted into pNN265 plasmid, with a modified multiple cloning site, that carries a 5' hybrid intron and a 3' intron plus poly-A signal from SV40 through PacI and SalI sites. Finally, the entire DNA fragment of HA-cEPOR, flanked by a hybrid intron at the 5' end and a polyadenylation signal from SV40 at the 3' end was cut out from pNN265 vector using NotI and placed downstream of the 8.5 kb α-CaMKII promoter.

The TG founders were produced by pronuclear injection of the linearized DNA into C57BL6/N *('TG1') *or FvB/N *('TG2') *zygotes. The analysis of line TG1 mice was performed after four to seven backcrosses with C57BL6/N wildtype mice (that is all results reported in this study were obtained from generations four to seven of the TG1 line). The TG1 line was used (because of its clean C57BL6/N background) for the behavioral experiments presented here. The analysis of line TG2 mice was performed after eight to nine backcrossings to C57BL6/N mice.

The genotype of transgenic offspring was analyzed by PCR of tail genomic DNA using primers specific for the 3' end of the α-CaMKII promoter sequence (5'-GGGAGGTAGGAAGAGCGATG-3') and the 5' end of the HA-cEPOR cDNA sequence (5'-CACCCTGAGTTTGTCCATCC-3') yielding a 769 bp product. PCR amplification of the tail DNA was carried out with the following conditions: 2 minutes, 94°C (1 cycle); 30 seconds 94°C, 30 seconds 60°C, 1 minute 72°C (35 cycles), followed by final extension at 72°C for 10 minutes.

#### Immunofluorescence

Wildtype and TG male HA-cEPOR mice were transcardially perfused under deep anesthesia with saline followed by 4% paraformaldehyde in 0.1 M sodium phosphate buffer (PBS) (pH 7.4). Brains were removed from the skulls, postfixed in 4% PFA overnight at 4°C and subsequently cryoprotected in 30% sucrose/PBS solution. After being frozen on dry ice, coronal cryosections (30 μm) were collected, washed briefly with PBS and incubated for 40 minutes at room temperature (RT) with 0.1% glycine (Merck, Darmstadt, Germany), 0.1% Triton X-100 in PBS. Sections were then immersed for one hour at RT in blocking solution [5% normal horse serum (NHS), 0.1% Triton X-100 in PBS] and incubated with mouse monoclonal anti-HA (1:500, Covance, Hiss Diagnostics, Freiburg, Germany) diluted in 3% NHS, 0.1% Triton X-100/PBS overnight at 4°C. After washing with PBS, sections were treated with anti-mouse Cy3-coupled secondary antibody (1:1000, Jackson ImmunoResearch Laboratories-Dianova, Hamburg, Germany) for one hour at RT. Following PBS washes, sections were mounted on Super Frost microscopic slides, air dried and coverslipped using Aqua-Poly/Mount (Polysciences, Eppelheim, Germany). Sections were imaged with a fluorescence stereomicroscope (Leica MZ16 FA, Wetzlar, Germany).

### Protein extraction and immunoblotting

Wildtype and TG HA-cEPOR male mice were sacrificed by cervical dislocation. The tissue samples were dissected and immediately frozen on dry ice. For immunoblotting, tissue samples including hippocampus, cortex, cerebellum, liver and kidney, were homogenized in lysis buffer (50 mM Tris HCL (pH 8.3), 150 mM NaCl, 40 mM NaF, 5 mM EDTA, 5 mM EGTA, 1 mM Na_3_VO_4_, 1% Igepal, 0.1% Natriumdesoxycholat, 0.1% SDS] containing 1 mM phenylmethysulfonylfluoride, 10 μg/ml aprotinin and 0.1 mg/ml leupeptin) using an Ultra-turrax homogenizer (Kinematica, Luzern, Switzerland). The lysates were centrifuged (1200 rpm) for 45 minutes at 4°C. The supernatant was collected and mixed with three volumes of Laemmli buffer [250 mM Tris HCL (pH 8.3), 8% SDS, 40% glycerol, 20% 2-mercaptoethanol, 0.04% pyronin Y], and boiled for ten minutes at 70°C for immunoblotting. The protein samples were run on NuPAGE 4 - 12% Bis-Tris Gel (Invitrogen, Karlsruhe, Germany) for one hour at 200 V and transferred to a nitrocellulose membrane. After blocking with 5% milk in Tween 20-Tris-buffered saline (TTBS) for one hour at RT, membranes were incubated in primary antibodies for rat monoclonal anti-HA (1:500, Roche, Mannheim, Germany) with mouse monoclonal anti-GAPDH (1:10000, Assay Designs/Stressgen, Ann Arbor, MI, USA) as an internal control. Immunoreactive bands were visualized using secondary antibodies coupled to horseradish peroxidase by enhanced chemoluminescence (Amersham, Freiburg, Germany).

### RNA isolation and expression analysis by reverse transcriptase PCR (RT-PCR)

Brains of E12, E17, P0, P14 and adult (two months old) TG male HA-cEPOR mice were rapidly frozen after being sacrificed. Tissue was homogenized in Trizol using an Ultra-turrax homogenizer (Kinematica, Luzern, Switzerland). Total RNA was isolated by using the RNeasyPlus kit (Qiagen, Hilden, Germany). During RNA isolation, on-column DNase digestion was performed with the RNase-free DNase set (Qiagen, Hilden, Germany). cDNA was prepared using N9 random and Oligo(dT)18 primers. HA-cEPOR expression was detected by PCR using the sense (5' CTACCCATACGACGTCCCAG 3') and antisense (5' GCGTCCAGGAGCACTACTTC 3') primers specific for the transgene, yielding a 362 bp product. GAPDH cDNA was amplified as internal control using the sense 5' TGCCAAGGCTGTGGGCAAGG 3' and antisense 5' TGTTGGGGGCCGAGTTGGGA 3' primers (431 bp). PCR amplification was done under the following conditions: 2 minutes, 94°C (1 cycle); 45 seconds 94°C, 45 seconds 58°C, 1 minute 72°C (30 cycles), followed by final extension at 72°C for 10 minutes.

### Slice preparation and electrophysiological recordings

Synaptic function and plasticity were assessed in acute brain tissue slices, with the experimenter blinded to the genotype under investigation. Adult male wildtype and TG HA-cEPOR mice (three to four months old) were decapitated under deep ether anesthesia, the brain was rapidly removed from the skull and placed in chilled artificial cerebrospinal fluid (ACSF) for one to two minutes. Acute neocortical/hippocampal tissue slices (400 μm thick transverse slices) were cut from the forebrain using a vibroslicer (752M Vibroslice, Campden Instruments, Loughborough, UK). The slices were then separated in the sagittal midline, transferred to an Oslo style interface recording chamber and left undisturbed for at least 90 minutes to ensure recovery from surgical trauma. The recording chamber was kept at a temperature of 32 - 33°C, continuously aerated with 95% O_2 _- 5% CO_2 _(400 ml/minute), and perfused with oxygenated ACSF (3 - 4 ml/minute). The ACSF contained (in mM): 130 NaCl, 3.5 KCl, 1.25 NaH_2_PO_4_, 24 NaHCO_3_, 1.2 CaCl_2_, 1.2 MgSO_4_, and 10 dextrose; aerated with 95% O_2 _- 5% CO_2 _to adjust pH to 7.4.

Orthodromically evoked field excitatory postsynaptic potentials (fEPSPs) were elicited by stimulation of Schaffer collaterals and recorded in *stratum radiatum *of the CA1 subfield with a locally constructed extracellular DC potential amplifier as described earlier [17,52]. Unipolar stimuli of 0.1 ms duration, negative polarity and 10-150 μA amplitude were generated by a stimulator (Grass S88 stimulator equipped with PSIU6 stimulus isolation units, Grass Instruments, Astro-Med Inc., Rodgau, Germany) and delivered via stimulation electrodes made from steel microwire (50 μm diameter, AM-Systems, Carlsborg WA, USA; [53]). Extracellular recording electrodes were pulled from thin-walled borosilicate glass capillaries (GC150TF-10, Harvard Apparatus, Holliston MA, USA) using a horizontal electrode puller (P-97 Flaming/Brown Micropipette Puller, Sutter Instruments, Novato CA, USA). They were filled with ACSF and their tips were trimmed to a final resistance of approximately 5 MΩ. Evoked responses were sampled at an acquisition rate of 20 kHz using an Axon Instruments Digitizer 1322A and PClamp 9.2 software (Molecular Devices Corporation, Sunnyvale CA, USA). Synaptic function and neuronal excitability were assessed by recording input-output curves (10-150 μA stimulus intensity) and synaptic plasticity was tested by inducing paired-pulse facilitation (PPF) and long-term potentiation (LTP). For PPF and LTP recordings, stimulation intensity was adjusted to obtain half-maximum response amplitudes. In PPF recordings, the inter-stimulus interval was varied in the range of 25 - 200 ms. LTP was induced by three 1- second lasting 100 Hz trains, separated by 20 seconds each, and fEPSPs were then recorded for 60 minutes. To improve the signal to noise ratio of the recordings, four consecutive sweeps were averaged online [53]; individual stimuli were delivered every 5 seconds (input-output curves and PPF) or 15 seconds (LTP). To quantify changes in synaptic function and plasticity, the amplitude of the EPSPs and their slope (within the 20% to 80% range of the falling phase) were analyzed using PClamp 9.2 software (Molecular Devices). To cancel out differences in absolute fEPSPs slopes and amplitudes arising from electrode positions and the distance of recordings and stimulation electrodes in individual slices, fEPSP amplitudes and slopes (normalized to maximum amplitude/slope measured in a given slice) were analyzed.

### Behavioral testing

All experiments were approved by the local Animal Care and Use Committee in accordance with the German Animal Protection Law. For behavioral testing, mice were housed in groups of three to five in standard plastic cages, food and water *ad libitum *(except for the five-choice training period, see below). The temperature in the colony room was maintained at 20 - 22°C, with a 12 hour light-dark cycle (light on at 7:00 am). Behavioral experiments were conducted by an investigator, blinded to the genotype, during the light phase of the day (between 8:00 am and 17:00 pm). The order of testing was as follows: Elevated plus maze, open field, hole board, rotarod, pre-pulse inhibition, social interaction, Morris water maze, the five-choice serial reaction time task and marble burying test. The age of mice at the beginning of testing was 11 to 12 weeks. Inter-test interval was at least one to two days.

#### Elevated plus maze

In this test of anxiety, mice were placed in the central platform, facing an open arm of the plus-maze (made of grey plastic with a 5 × 5 cm central platform, 30 × 5 cm open arms and 30 × 5 × 15 cm closed arms; illumination 120 lx). The behavior was recorded for five minutes by an overhead video camera and a PC equipped with "Viewer 2" software (Biobserve GmbH, Bonn, Germany) to calculate the time spent in open or closed arms, distance traveled, number of arm visits, and velocity. The proportion of time spent in open arms was used to estimate open arm aversion (fear equivalent).

#### Open field

Spontaneous activity in the open field was tested in a grey Perspex arena (120 cm in diameter, 25 cm high; illumination 120 lx). Mice were placed in the center and allowed to explore the open field for seven minutes. The behavior was recorded by a PC-linked overhead video camera. "Viewer 2" software was used to calculate velocity, distance traveled, and time spent in central, intermediate or peripheral zones of the open field.

#### Hole board

The hole board test measures exploratory activity. The apparatus consisted of a 51 × 51 × 33 cm transparent Perspex chamber with a non-transparent floor, with 16 equally spaced holes, 2 cm in diameter, 2 cm deep. Mice were allowed to explore the chamber for five minutes and the number of explored holes (head dips) was registered by a computer software (TSE GmbH, Bad Homburg, Germany). The illumination in the testing room was 120 lx.

#### Rotarod

The rotarod test examines motor function, balance, and coordination. It comprised a rotating drum (Ugo Basile, Comerio, Varese, Italy), which was accelerated from 4 to 40 rpm over five minutes. Mice were placed individually on the drum and the latency of falling off the drum was recorded using a stop-watch. To assess motor learning, the rotarod test was repeated 24 hours later.

#### Pre-pulse inhibition test

In this test of sensorimotor gating, individual mice were placed in small metal cages (90 × 40 × 40 mm) to restrict major movements and exploratory behavior. The cages were equipped with a movable platform floor attached to a sensor that records vertical movements of the floor. The cages were placed in four sound-attenuating isolation cabinets (TSE GmbH, Bad Homburg, Germany). Startle reflexes were evoked by acoustic stimuli delivered from a loudspeaker that was suspended above the cage and connected to an acoustic generator. The startle reaction to an acoustic stimulus, which evokes a movement of the platform and a transient force resulting from this movement of the platform, was recorded with a computer during a recording window of 260 ms (beginning with the onset of pre-pulse) and stored for further evaluation. The recording window was defined from the onset of the acoustic stimulus. An experimental session consisted of a two minute habituation to 65 dB background white noise (continuous throughout the session), followed by a baseline recording for one minute at background noise. After baseline recording, six pulse-alone trials using startle stimuli of 120 dB intensity and 40 ms duration were applied in order to decrease influence of within-session habituation. These data were not included in the analysis of the pre-pulse inhibition. For tests of pre-pulse inhibition, the 120 dB/40 ms startle pulse was applied either alone or preceded by a pre-pulse stimulus of 70 db, 75 db, or 80 dB sound pressure level and 20 ms duration. An interval of 100 ms with background white noise was employed between each pre-pulse and pulse stimulus. The trials were presented in a pseudorandom order with an interval ranging from 8 to 22 seconds. The amplitude of the startle response (expressed in arbitrary units) was defined as the difference between the maximum force detected during a recording window and the force measured immediately before the stimulus onset. Amplitudes were averaged for each individual animal, separately for both types of trials (that is stimulus alone or stimulus preceded by a pre-pulse). Pre-pulse inhibition was calculated as the percentage of the startle response using the following formula: *% pre-*pulse inhibition = *100 - *[(startle amplitude after pre-pulse and pulse)/(startle amplitude after pulse only) × 100].

#### Social interaction

Sociability and social memory were tested as described in detail elsewhere (for example [54]). The social testing arena was a rectangular, three-chambered box. Each chamber was 20 × 40 × 22 cm in size. Dividing walls were made from clear Plexiglas, with rectangular openings (35 × 35 mm) allowing access into each chamber. The chambers of the arena were cleaned, and fresh paper chip bedding was added between trials. The test mouse was first placed in the middle chamber and allowed to explore for five minutes. The openings into the two-side chambers were obstructed by plastic boxes during this habituation phase. After the habituation period, an unfamiliar C57BL/6NCrl male mouse (stranger one) without prior contact with the subject mouse was placed in one of the side chambers. The location of stranger one in the left versus right side chamber was systematically alternated between trials. The stranger mouse was enclosed in a small (60 × 60 × 100 mm), rectangular wire cage, which allowed nose contact through the bars but prevented fighting. The animals serving as strangers had previously been habituated to placement in the small cage. An identical empty wire cage was placed in the opposite chamber. A heavy cup was placed on the top of each of the small wire cages to prevent climbing by the test mice. Both openings to the side chambers were then unblocked, and the subject mouse was allowed to explore the entire social test arena for a ten minute session. The amount of time spent in each chamber and the number of entries into each chamber were recorded by the video tracking system "Viewer 2" (Biobserve GmbH). An entry was defined as all four paws in one chamber. At the end of the first ten minute trial, each mouse was tested in a second ten minute session to quantify social preference for a new stranger. A second, unfamiliar mouse (stranger two) was placed into the previously empty wire cage. The test mouse had a choice between the first, already explored mouse (familiar stranger one), and the novel unfamiliar mouse (new stranger two). As described above, measures were taken of the amount of time spent in each chamber and the number of transitions between chambers of the apparatus during the second ten minute session. Based on the amount of time spent in each chamber, a 'sociability index' and a 'social memory index' (with a value of 0 meaning no preference) were calculated according to the following formulas:

#### Morris water maze

Spatial learning and memory was assessed in a water maze [27]. A large circular tank (diameter 1.2 m, depth 0.4 m) was filled with opaque water (25 ± 1°C, depth 0.3 m) and the escape platform (10 × 10 cm) was submerged 1 cm below the surface. The swim patterns were monitored by a computer and the video-tracking system "Viewer 2". The escape latency, swim speed, path length, and trajectory of swimming were recorded for each mouse. During the first two days, mice were trained to swim to a clearly visible platform (visible platform task) that was marked with a 15 cm high black flag and placed pseudo-randomly in different locations across trials (non-spatial training). The extra-maze cues were hidden during these trials. After two days of visible platform training, hidden platform training (spatial training) was performed. For eight days, mice were trained to find a hidden platform (that is the flag was removed) that was located in the center of one of the four quadrants of the pool. The location of the platform was fixed throughout testing. Mice had to navigate using extra-maze cues that were placed on the walls of the testing room. Every day, mice went through four trials with an inter-trial interval of five minutes. The mice were placed into the pool facing the side wall randomly at one of four start locations and allowed to swim until they found the platform, or for a maximum of 90 seconds. Any mouse that failed to find the platform within 90 seconds was guided to the platform. The animal then remained on the platform for 20 seconds before being removed from the pool. The next day after completion of the hidden platform training, a probe trial was conducted in order to determine whether mice used a spatial strategy to find the platform or not. The platform was removed from the pool and the mice were allowed to swim freely for 90 seconds. The percentage of time spent in each quadrant of the pool as well as the number of times the mice crossed the former position of the hidden platform were recorded. In order to investigate the flexibility of cognitive processes in mice, the reversal water maze test was performed. The experimental procedure was identical to the one used for the hidden platform training with the exception that the escape platform was moved from the original position to the neighboring quadrant.

#### The 5-choice serial reaction time task (5-CSRTT)

The 5-choice serial reaction time task (5-CSRTT) measures higher brain functions, ranging from various discrete learning/memory to attentional paradigms [55,56]. Mice were trained in an operant chamber (width 15.5 cm, depth 20 cm, height 18 cm; Med Associates Inc, St. Albans, USA), enclosed in a sound attenuating box and connected to a Fujitsu Siemens PC. One wall of the operant chamber had a curved shape and carried an array of five stimulus holes. The stimulus holes were 1.2 cm in diameter and contained a LED stimulus light (depth: 1 cm) in the rear. Infrared photocell pairs were located at 4 mm from the entrance of the stimulus holes and detected nose pokes of mice into the holes. The wall opposite to the stimulus holes contained a magazine cup, also with a photocell detector of head entries, in which liquid reward (4% sucrose solution) was delivered always simultaneously with illumination of the magazine. The house light was located 32 cm above the magazine.

##### Habituation and magazine training

Two days before starting training, mice were habituated to the liquid reward of 4% sucrose solution in their home cages over night. The day before starting magazine training, sucrose bottles were removed and mice were water deprived. Water deprivation was applied during the entire experimental period. Immediately after finishing the daily test sessions, mice were given water in individual cages for 20 minutes. Magazine training consisted of four consecutive phases (M1 to M4), one phase per day, each lasting for 15 minutes, with all stimulus holes closed. In the first phase (M1), liquid reward was delivered (10 μl) upon initiation of the training session. In the second phase of magazine training (M2), the number of potential rewards was increased, with a fixed interval of 118 seconds between reward presentations. A head entry into the magazine was required to collect the reward. In the third phase (M3), the fixed interval was replaced by a head entry-dependent interval of 100 seconds to obtain reward. In the last phase (M4), this interval was further reduced to 50 seconds, ideally yielding a consistently increasing number of head entries. Head entries into the magazine together with reward consumption were taken as indicators for associating the magazine with reward delivery.

##### Shaping phases (operant and discriminant learning)

During shaping, mice were trained to perform a nose poke into an illuminated stimulus hole in order to obtain reward. The shaping procedure consisted of two phases (each extending over several days, dependent on individual performance, and with a daily session duration of 30 minutes) where mice were taught to associate nose poking into an illuminated hole with reward (phase S1), and then trained to discriminate between those nose pokes that lead to reward (illuminated holes) and those that do not (unlit holes) (phase S2). Throughout shaping all stimulus holes were open. During S1, all stimulus lights were on. Any nose poke in a stimulus hole was rewarded. The inter-trial interval (time from pick-up of reward to next stimulus hole illumination) was set to eight seconds. During S2, presentation of lit and unlit holes was conducted in a pseudorandom manner. Mice were only rewarded upon nose poking into a lit stimulus hole. Performing a nose poke in an unlit hole led to switch-off of the house light for five seconds. Mice in S1 were moved to the next training phase once they had reached 35 to 40 nose pokes each on three consecutive days. The number of trials in S2 was 60 per day and training in this phase was terminated when mice had arrived at a stable performance of ≥70% correct responses for three consecutive days.

##### 5-CSRTT training

The training session started with illumination of magazine light and presentation of 4% sucrose solution. Head entry started the trial. At eight seconds after head entry, light (initially set to sixteen seconds) was randomly presented in one of the five stimulus holes. A correct response, that is nose poking into the lit hole, led to reward (6 μl) and the next trial start after eight seconds (inter-trial interval, ITI). Nose poking in an unlit stimulus hole, that is an incorrect response, led to extinguishing the house light for five seconds (time-out) and no reward. Further nose pokes during time-out extended that period for an additional five seconds each. If a mouse did not respond by nose poking into any of the holes during stimulus presentation, an omission was counted. As a consequence, no reward was presented. Also omissions provoked time-out. A training session was terminated after 30 minutes or upon performing 60 trials, whichever came first. Mice were trained in the phase with 16 second stimulus duration until they reached clearly defined performance criteria (≥75% accuracy [correct responses/correct + incorrect responses * 100], ≤20% omissions and at least 50 trials performed over three consecutive days). Six such phases followed with gradually declining stimulus duration up to 1.4 seconds (16, 8, 4, 2, 1.8 and 1.4 seconds). In the first phases, mice had time to respond as long as the stimulus light was on. For phases with stimulus duration below five seconds, the response time (so called limited hold) was added up to five seconds.

##### Baseline training and attentional manipulations

After the acquisition phase, which was terminated by reaching stable performance criteria in the 1.4 second-phase (see above), mice were trained at 'baseline parameters', that is stimulus duration of 1.4 seconds, an ITI of 8 seconds and a maximum of 60 trials. This training was performance-independent, lasted for 21 days (one session/day) and was conducted to further stabilize performance of mice [57]. Immediately after baseline training, attentional intervention phases (INT1-5) succeeded in the following manner: (INT1) variable, long ITI (8, 9, 10 and 11 second); (INT2) variable, short ITI (5, 6, 7 and 8 second); (INT3) variable, short stimulus duration (1.4, 1.0, 0.6 and 0.2 seconds); (INT4) variable long ITI (8, 9, 10 and 11 seconds) coupled with variable short stimulus duration (1.4, 1.0, 0.6 and 0.2 seconds) and (INT5) short stimulus duration (0.6 second) applied simultaneously with a sound distracter (80 dB, white noise). INT1, INT2, INT3 and INT5 lasted for four days (one session/day), while INT4 was conducted for 15 days (one session/day). Between two intervention phases, mice were kept in a 'recovery' phase, consisting of baseline parameters, until they re-reached the above described baseline performance criteria. This was done to rule out the impact of motivational and learning factors resulting from the prior intervention phase [58]. Main task parameters analyzed in all experimental conditions were the following: % of omissions (= proportion of omitted trials), % of accuracy (= proportion of correct responses), reward latency, reaction time and number of premature responses. All these parameters, except for the number of premature responses, were assessed over all trial blocks (that is one trial block contains ten trials, six trial blocks in total) and/or over the corresponding inter-trial intervals, and/or the respective stimulus durations, collapsed over the whole corresponding test period. For premature responses, the average over the corresponding test period was calculated.

#### Marble burying test

The marble burying test is used to assess stereotypies and obsessive-compulsive behaviors in mice [59]. Mice were tested in plastic cages (34.5 × 56.5 × 18 cm) filled with 5 cm deep wood chip bedding. Twenty-four glass marbles evenly spaced (4 cm apart) were placed on the surface. Individual mice were put in the cage and left there for 30 minutes. Illumination was dimmed (6 lx). The number of buried marbles (to 2/3 their depth) during this time was counted.

### Statistical analysis

Statistical analysis was performed using the statistical programs SPSS for windows, release 16 (SPSS Inc., Chicago, USA) and GraphPad Prism version 4.00 for Windows, (GraphPad Software, San Diego, USA). We applied 2-way ANOVA for repeated measures, chi^2^-Test, Mann-Whitney U-Test, Wilcoxon Test and survival analysis where indicated. Bonferroni and Dunn's multiple comparison tests were used for post-hoc analysis. Threshold for significance was *P *< 0.05. All data are presented as mean ± SEM (standard error of the mean).

## Abbreviations

α-CaMKII: α-calcium/calmodulin-dependent protein kinase II; ACSF: Artificial cerebrospinal fluid; cEPOR: Constitutively active EPOR; ERK 1/2: RAS/extra-cellular signal-regulated kinase; EPO: Erythropoietin; EPOR: Erythropoietin receptor; fEPSPs: Field excitatory postsynaptic potentials; fMRI: Functional magnetic resonance imaging; HA: Hemagglutinin; INT 1-5: Intervention phases 1-5; ITI: Inter-trial interval; LTP: Long-term potentiation; M 1-4: Magazine training phases 1-4; NF-kappa B: Nuclear factor kappa B; (PI3K)/AKT: Phosphatidylinositol-3 kinase; PPF: Paired-pulse facilitation; PTP: Post-tetanic potentiation; rhEPO: Recombinant human erythropoietin; RT: Room temperature; S1-2: Shaping phases 1-2; STATs: Signal transducers and activators of transcription; TG: Transgenic; WT: Wildtype; 5-CSRTT: 5-choice serial reaction time task

## Competing interests

User patents on EPO in schizophrenia and MS have been submitted.

## Authors' contributions

DS and AA generated the transgenic mice. DS, SS and IH conducted and analyzed all molecular biological experiments. AEK performed all behavioral experiments and statistical analysis of behavioral and electrophysiological results. MM carried out the electrophysiological recordings and analyzed the respective data. SMW participated in the conceptual work of the study. KAN and HE designed, coordinated and supervised the study. AEK, DS and HE participated in preparing and writing the manuscript. All authors read and approved the final manuscript.

## Supplementary Material

Additional file 1 **Expression of HA-cEPOR in the *'TG2' *line**. Forebrain-specific expression of HA-cEPOR transgene also in line *'TG2' *was revealed by immunohistochemistry. A monoclonal antibody against the HA-tag was used to stain coronal sections of hippocampus. HA-cEPOR expression is absent in WT mice (A and B). Similar to the line *'TG1'*, HA-cEPOR expression is restricted to the pyramidal neurons of cortex (C), CA1 (D), CA3 subregions of hippocampus and granular layer of dentate gyrus. Scale bars; 100 μm and 500 μm. Click here for file

Additional file 2 **Overexpression of cEPOR does not affect overall brain morphology**. (A) Coronal sections from WT and TG mice were stained with haematoxylin-eosin and (B) Luxol Fast Blue. General brain morphology and myelin architecture were comparable between WT and TG mice. Scale bars; 1 mm. Click here for file

Additional file 3 **Within-day trial analysis in the hidden platform paradigm of Morris water maze**. In cEPOR TG mice, there is a tendency of faster within-day learning, that is shorter latency to reach the platform on days one and two. However, on most of the training days, ANOVA did not reach significance levels. WT n = 18; TG n = 13; mean ± SEM presented. Click here for file

Additional file 4 **Main parameters and results of the 5-choice serial reaction time task (5-CSRTT) in WT and cEPOR TG mice**. Click here for file
