# Supplementary figures and images for: Expression of constitutively active erythropoietin receptor in pyramidal neurons of cortex and hippocampus boosts higher cognitive functions in mice
Source: BMC Biol. 2011 Apr 28;9:27. doi: 10.1186/1741-7007-9-27 (PMC3120735; doi:10.1186/1741-7007-9-27)

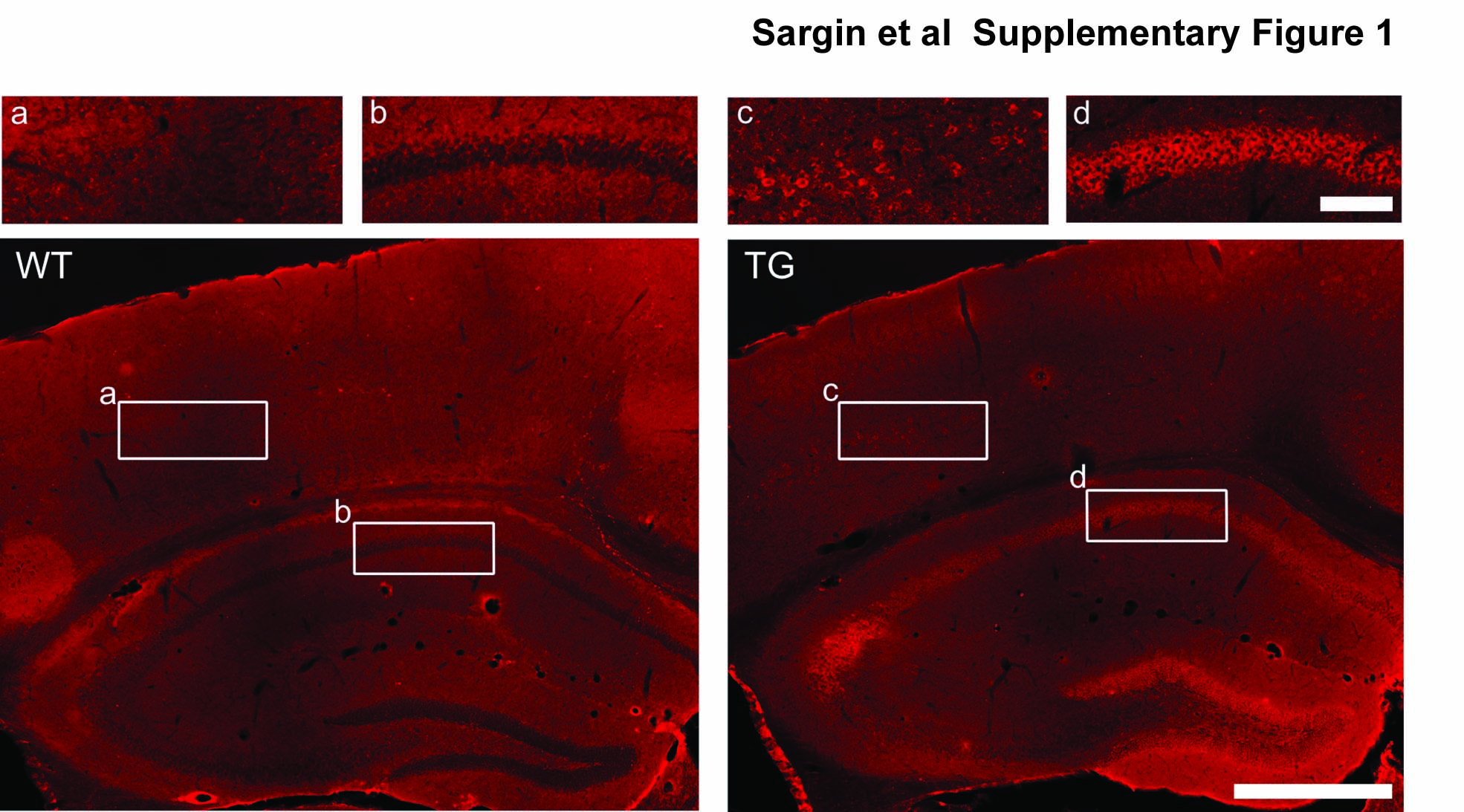

Supplement: Additional file 1 — Expression of HA-cEPOR in the 'TG2' line. Forebrain-specific expression of HA-cEPOR transgene also in line 'TG2' was revealed by immunohistochemistry. A monoclonal antibody against the HA-tag was used to stain coronal sections of hippocampus. HA-cEPOR expression is absent in WT mice (A and B). Similar to the line 'TG1', HA-cEPOR expression is restricted to the pyramidal neurons of cortex (C), CA1 (D), CA3 subregions of hippocampus and granular layer of dentate gyrus. Scale bars; 100 μm and 500 μm. [file 1741-7007-9-27-S1.JPEG]

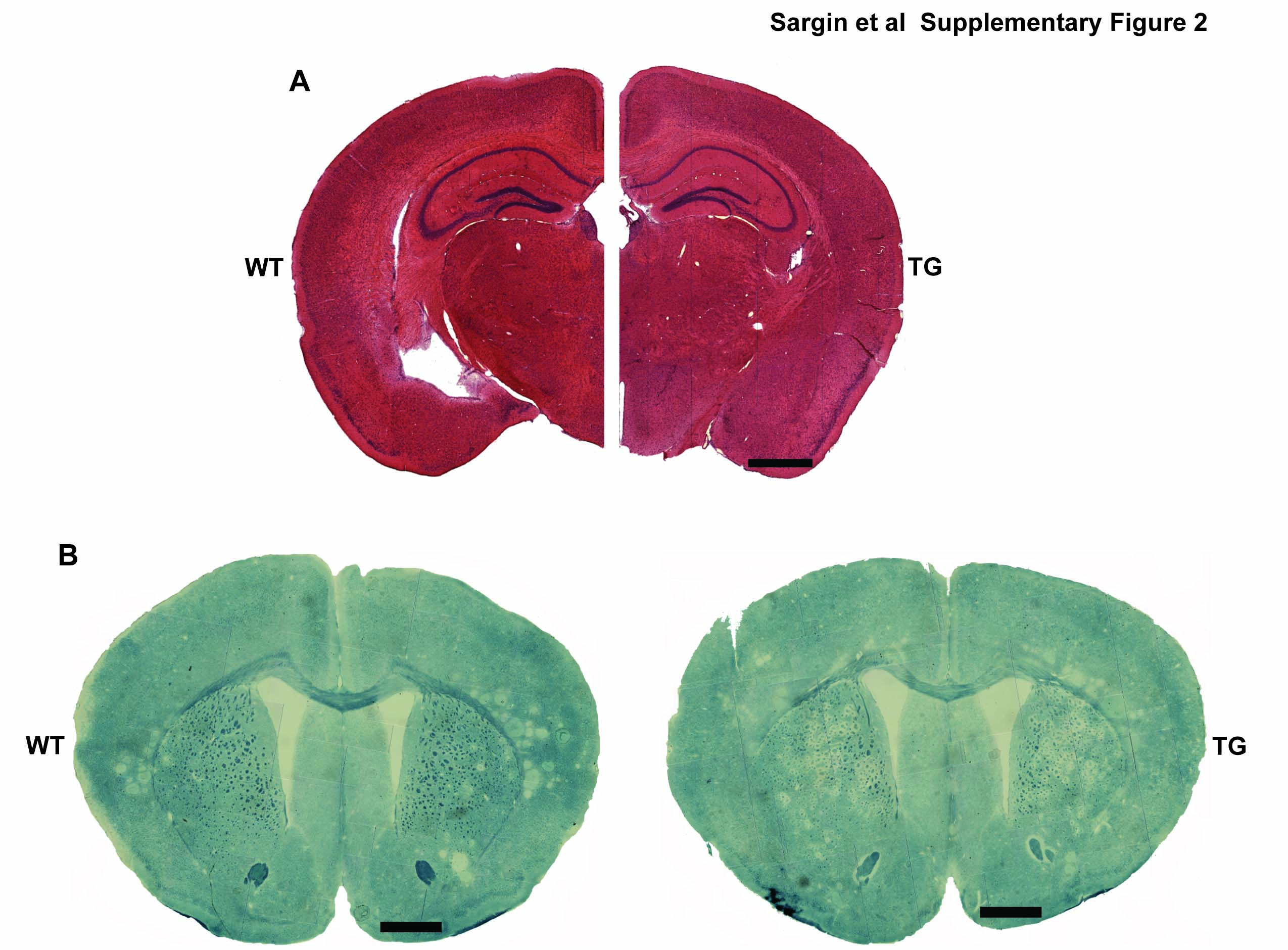

Supplement: Additional file 2 — Overexpression of cEPOR does not affect overall brain morphology. (A) Coronal sections from WT and TG mice were stained with haematoxylin-eosin and (B) Luxol Fast Blue. General brain morphology and myelin architecture were comparable between WT and TG mice. Scale bars; 1 mm. [file 1741-7007-9-27-S2.JPEG]

# Sargin et al Supplementary Figure3

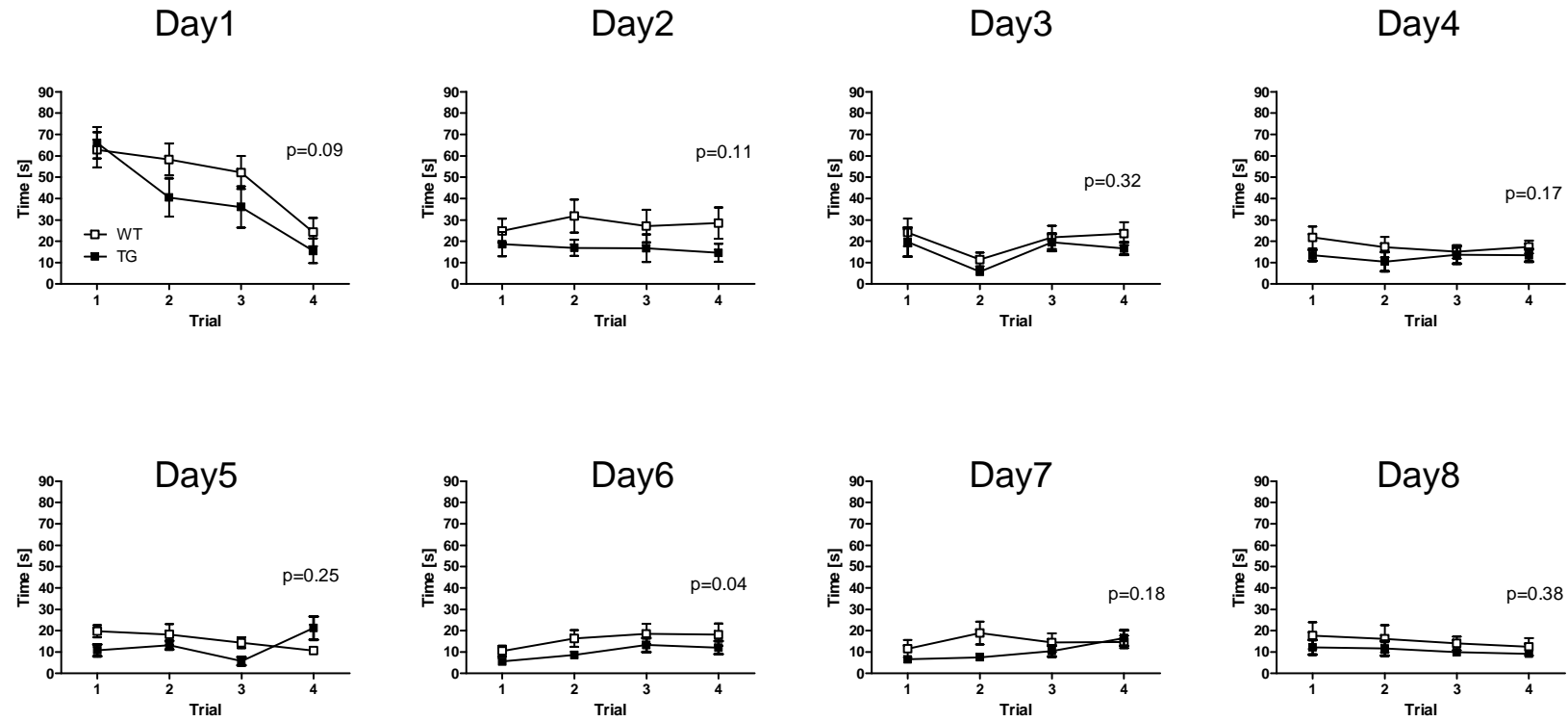

Supplement: Additional file 3 — Within-day trial analysis in the hidden platform paradigm of Morris water maze. In cEPOR TG mice, there is a tendency of faster within-day learning, that is shorter latency to reach the platform on days one and two. However, on most of the training days, ANOVA did not reach significance levels. WT n = 18; TG n = 13; mean ± SEM presented. [file 1741-7007-9-27-S3.PDF]
