# Supplementary material for: Expression of constitutively active erythropoietin receptor in pyramidal neurons of cortex and hippocampus boosts higher cognitive functions in mice
Source: BMC Biol. 2011 Apr 28;9:27. doi: 10.1186/1741-7007-9-27 (PMC3120735; doi:10.1186/1741-7007-9-27)
Supplement: Additional file 4 — Main parameters and results of the 5-choice serial reaction time task (5-CSRTT) in WT and cEPOR TG mice. [file 1741-7007-9-27-S4.DOC]

**Supplementary Table S**1

1

|  |  |  |  |  | **Results** | | | |
| --- | --- | --- | --- | --- | --- | --- | --- | --- |
| **Testing phase** | **Task characteristics** | **Parameter** | **Presentation of data** | **Condition** | **Mean**  **WT TG** | | **95% Confidence interval** | **Test statistics^*^** |
| Baseline training | Stimulus duration: 1.4s  Inter-trial interval: 8s  60 trials/day, 21days | Accuracy  (%) | Not shown | 1^A^ | 95.42 | 94.78 | -3.412 to 2.130 | F_(1,16)_=1.940  p=0.183 |
|  |  |  |  | 2 ^A^ | 97.02 | 97.67 | -2.127 to 3.415 |  |
|  |  |  |  | 3 ^A^ | 96.22 | 96.92 | -2.075 to 3.467 |  |
|  |  |  |  | 4 ^A^ | 96.08 | 97.87 | -0.9759 to 4.566 |  |
|  |  |  |  | 5 ^A^ | 95.92 | 97.77 | -0.9168 to 4.625 |  |
|  |  |  |  | 6 ^A^ | 96.48 | 97.39 | -1.870 to 3.672 |  |
|  |  | Reaction time  (s) | Figure 4B | 1^A^ | 1.035 | 0.8557 | -0.2865 to -0.07296 | F_(1,16)_=10.730  p= 0.005 |
|  |  |  |  | 2 ^A^ | 0.9482 | 0.8429 | -0.2121 to 0.001459 |  |
|  |  |  |  | 3 ^A^ | 0.9391 | 0.8529 | -0.1930 to 0.02055 |  |
|  |  |  |  | 4 ^A^ | 0.9209 | 0.8600 | -0.1677 to 0.04587 |  |
|  |  |  |  | 5 ^A^ | 0.9264 | 0.8529 | -0.1803 to 0.03328 |  |
|  |  |  |  | 6 ^A^ | 0.9400 | 0.8571 | -0.1896 to 0.02393 |  |
| INT1  (variable, long inter-trial interval) | Stimulus duration:1.4s  Inter-trial interval: 8, 9, 10, 11s (randomized)  60 trials/day, 4days | Accuracy  (%) | Not shown | 8s^B^ | 96.44 | 96.53 | -4.806 to 4.978 | F_(1,12)_=0.328  p=0.578 |
|  |  |  |  | 9s^B^ | 94.73 | 96.70 | -2.923 to 6.861 |  |
|  |  |  |  | 10s^B^ | 94.66 | 95.82 | -3.732 to 6.052 |  |
|  |  |  |  | 11s^B^ | 96.61 | 96.60 | -4.908 to 4.876 |  |
|  |  | Reaction time  (s) |  | 8s^B^ | 0.9657 | 0.8700 | -0.2563 to 0.06487 | F_(1,12)_=3.411  p=0.089 |
|  |  |  |  | 9s^B^ | 0.9557 | 0.8014 | -0.3149 to 0.006296 |  |
|  |  |  |  | 10s^B^ | 0.8571 | 0.7714 | -0.2463 to 0.07487 |  |
|  |  |  |  | 11s^B^ | 0.9057 | 0.8471 | -0.2192 to 0.1020 |  |
| INT2  (variable, short inter-trial interval) | Stimulus duration: 1.4s  Inter-trial interval: 5, 6, 7, 8s (randomized)  60 trials/day, 4days | Accuracy  (%) | Not shown | 5s^B^ | 96.47 | 97.98 | -2.483 to 5.490 | F_(1,14)_=2.478  p=0.138 |
|  |  |  |  | 6s^B^ | 96.51 | 98.39 | -2.105 to 5.867 |  |
|  |  |  |  | 7s^B^ | 97.13 | 97.93 | -3.187 to 4.785 |  |
|  |  |  |  | 8s^B^ | 95.36 | 98.64 | -0.6989 to 7.274 |  |
|  |  | Reaction time  (s) | Figure 4C | 5s^B^ | 1.176 | 0.9271 | -0.3912 to -0.1057 | F_(1,14)_=10.420  p=0.006 |
|  |  |  |  | 6s^B^ | 1.062 | 0.8886 | -0.3164 to -0.03091 |  |
|  |  |  |  | 7s^B^ | 0.9878 | 0.8657 | -0.2648 to 0.02068 |  |
|  |  |  |  | 8s^B^ | 0.9178 | 0.8471 | -0.2134 to 0.07211 |  |
|  |  | Premature responses  (#) | Figure 4D | 5s^B^ | 0.6389 | 0.2143 | -1.010 to 0.1605 | F_(1,14)_=6.658  p=0.022 |
|  |  |  |  | 6s^B^ | 0.6389 | 0.1786 | -1.045 to 0.1248 |  |
|  |  |  |  | 7s^B^ | 0.7222 | 0.3571 | -0.9502 to 0.2201 |  |
|  |  |  |  | 8s^B^ | 0.9167 | 0.5000 | -1.002 to 0.1685 |  |

**Main parameters and results of the 5-choice serial reaction time task (5-CSRTT) in WT and cEPOR TG mice**

**Supplementary Table S1 *(continued)***

2

|  |  |  |  |  | **Results** | | | |
| --- | --- | --- | --- | --- | --- | --- | --- | --- |
| **Testing phase** | **Task characteristics** | **Parameter** | **Presentation of data** | **Condition** | **Mean**  **WT TG** | | **95% Confidence interval** | **Test statistics^*^** |
| INT3  (variable, short stimulus duration) | Stimulus duration: 0.2, 0.6, 1.0, 1.4s (randomized)  Inter-trial interval: 8s  60 trials/day, 4days | Accuracy  (%) | Not shown | 0.2s^C^ | 77.33 | 83.72 | -2.989 to 15.75 | F_(1,14)_=0.202  p=0.66 |
|  |  |  |  | 0.6s^C^ | 91.38 | 90.22 | -10.53 to 8.208 |  |
|  |  |  |  | 1.0s^C^ | 95.38 | 96.60 | -8.150 to 10.59 |  |
|  |  |  |  | 1.4s^C^ | 97.59 | 95.26 | -11.70 to 7.038 |  |
|  |  | Reaction time  (s) |  | 0.2s^C^ | 0.680 | 0.6586 | -0.1807 to 0.1379 | F_(1,14)_=4.180  p=0.060 |
|  |  |  |  | 0.6s^C^ | 0.7467 | 0.6329 | -0.2731 to 0.04549 |  |
|  |  |  |  | 1.0s^C^ | 0.8533 | 0.7157 | -0.2969 to 0.02168 |  |
|  |  |  |  | 1.4s^C^ | 0.9467 | 0.8371 | -0.2688 to 0.04977 |  |
| INT4  (variable, long inter-trial interval, variable short stimulus duration) | Stimulus duration: 0.2, 0.6, 1.0, 1.4s (randomized)  Inter-trial interval: 8, 9, 10, 11s (randomized)  64 trials/day, 15days | Accuracy  (%) | Not shown | 8s^B^ | 92.08 | 93.46 | -4.646 to 7.401 | F_(1,14)_=0.022  p=0.885 |
|  |  |  |  | 9s^B^ | 91.28 | 91.72 | -5.582 to 6.465 |  |
|  |  |  |  | 10s^B^ | 91.91 | 92.00 | -5.934 to 6.114 |  |
|  |  |  |  | 11s^B^ | 91.49 | 90.78 | -6.738 to 5.309 |  |
|  |  | Reaction time  (s) | Not shown | 8s^B^ | 0.8033 | 0.7143 | -0.2065 to 0.02838 | F_(1,14)_=2.728  p=0.121 |
|  |  |  |  | 9s^B^ | 0.7900 | 0.7000 | -0.2074 to 0.02743 |  |
|  |  |  |  | 10s^B^ | 0.7622 | 0.7071 | -0.1725 to 0.06235 |  |
|  |  |  |  | 11s^B^ | 0.7633 | 0.7200 | -0.1608 to 0.07410 |  |
|  |  | Omissions  (%) | Figure 5A | 8s^B^ | 37.41 | 39.25 | -6.514 to 10.20 | F_(1,14)_=1.849  p=0.195 |
|  |  |  |  | 9s^B^ | 34.95 | 38.08 | -5.230 to 11.48 |  |
|  |  |  |  | 10s^B^ | 35.88 | 39.51 | -4.722 to 11.99 |  |
|  |  |  |  | 11s^B^ | 35.00 | 42.16 | -1.194 to 15.52 |  |
| INT5  (short stimulus duration, sound distracter) | Stimulus duration: 0.6s  Inter-trial interval: 8s  Sound distracter duration: 0.6s, simultaneous onset with the light stimulus  (sound and no-sound trials are randomized)  60 trials/day, 4days | Accuracy  (%) | Figure 4F | No-sound | 93.60 | 90.45 | WT: -18.66108 to -3.53492  TG: -8.14630 to 8.63005 | Wilcoxon  WT: W=41.00, p=0.012  TG: W=-2.00, p=0.937 |
|  |  |  |  | Sound | 82.50 | 90.69 |  |  |
|  |  | Reaction time  (s) | Figure 4G | No-sound | 0.8327 | 0.7267 | -0.2646 to 0.05245 | F_(1,14)_=4.314  p=0.057 |
|  |  |  |  | Sound | 0.9307 | 0.7925 | -0.2967 to 0.02029 |  |
|  |  | Premature responses  (#) | Figure 5B | Overall average | 2.528 | 3.750 | WT: 1.8320 to 3.2236  TG: 2.8156 to 4.6844 | Mann-Whitney U=11.50  p=0.038 |

**Main parameters and results of the 5-choice serial reaction time task (5-CSRTT) in WT and cEPOR TG mice**

^A^ Data are analyzed over trial blocks (i.e. 10 trials per block) collapsed over 21 days; ^B^ Data are analyzed over inter-trial intervals; ^C^Data are analyzed over stimulus durations.

^*^Test statistics of 2-way ANOVA repeated measures if not otherwise specified.
